# Isolated congenital facial nerve agenesis

**DOI:** 10.1259/bjrcr.20220119

**Published:** 2023-05-15

**Authors:** Amarit Kay Gill, Ashok Raghavan, Eishaan Kamta Bhargava

**Affiliations:** 1 Sheffield Teaching Hospitals NHS Foundation Trust, Sheffield, England; 2 University of Sheffield, Sheffield, United Kingdom; 3 Sheffield Children's Hospital NHS Foundation Trust, Sheffield, England

## Abstract

An otherwise healthy 2-month-old boy was referred to ENT for a congenital right facial palsy, with a birth history of difficult ventouse delivery. Initially, a traumatic cause was suspected, however subsequent MR 3D-FIESTA (*T_2_* weighted) imaging demonstrated a right facial nerve agenesis with normal appearances of the remainder of the brain parenchyma, cranial nerves and parotid glands. There were no syndromic features or hearing difficulties. Isolated congenital nerve agenesis is a rare condition, with very few case reports available in the literature. Pre-natal 4D ultrasound imaging further supports the diagnosis. To our knowledge, this is the first published pre-natal ultrasound image of congenital facial nerve palsy. The infant has been referred for consideration of nerve reconstruction surgery, and is receiving multi-disciplinary input from ENT, Physiotherapy and Ophthalmology, the latter for prevention of exposure keratitis.

## Background

Isolated congenital facial nerve agenesis is a rare condition, particularly in the absence of associated hearing difficulties or syndromic features. There are very few case reports currently available in the literature. Congenital facial paralysis is also associated with a history of traumatic birth, and it is therefore important to recognise the condition early and distinguish the underlying cause, as this may alter management, prognosis, and potential medico-legal implications. This case study aims to strengthen existing literature due to the rarity of the condition.

## Case presentation

A 2-month-old boy was referred to ENT for a suspected traumatic right facial palsy. Birth history included a difficult ventouse delivery. There was no history of infection. Following birth, he had an inability to close the right eye with a downward turn of the right angle of the mouth when crying. There were no concerns about hearing or feeding. Clinical examination revealed a House-Brackmann Grade 2/3 facial paresis with no observed asymmetry at rest and intact Bell’s phenomenon. Remainder of the ENT and cranial nerve examination was unremarkable, and there were no features suggestive of Moebius syndrome.

## Investigations

**Figure 1.**
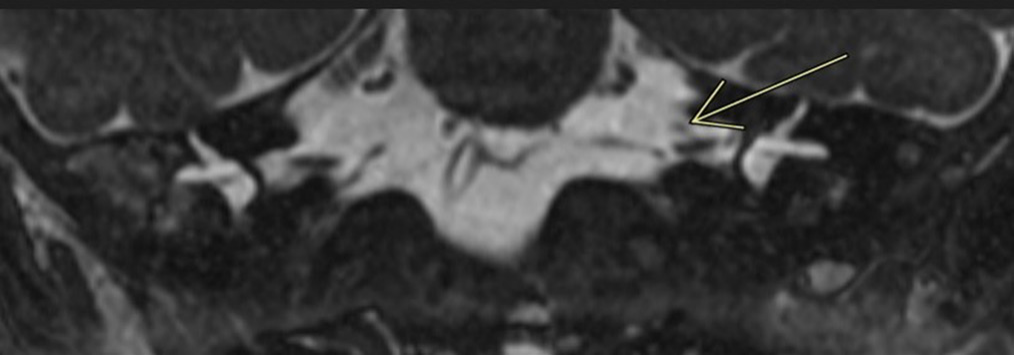
3D-FIESTA (*T_2_* weighted) coronal reconstruction depicting absence of the right seventh cranial nerve. The left seventh cranial nerve is identified (arrow).

**Figure 2.**
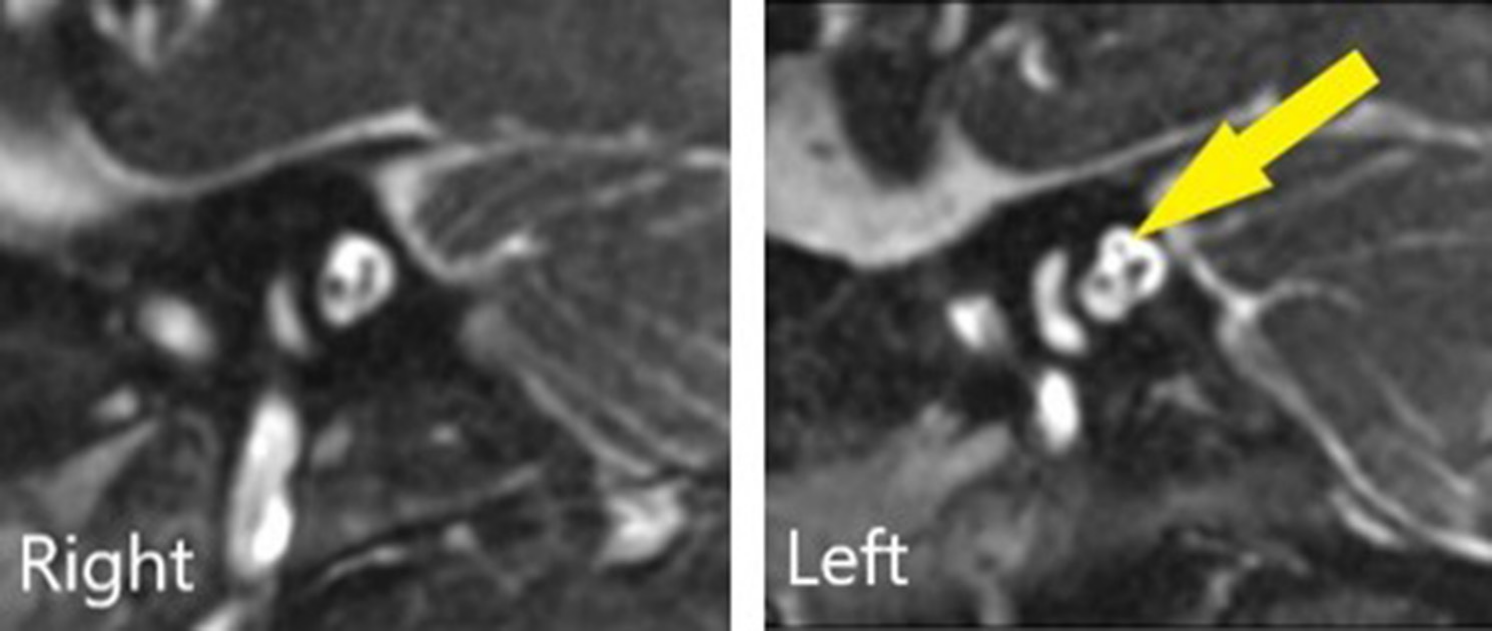
3D-FIESTA (*T_2_* weighted) sagittal reconstructions, depicting the normal left seventh cranial nerve (yellow arrow) and absent right seventh cranial nerve within the internal auditory canals.

**Figure 3.**
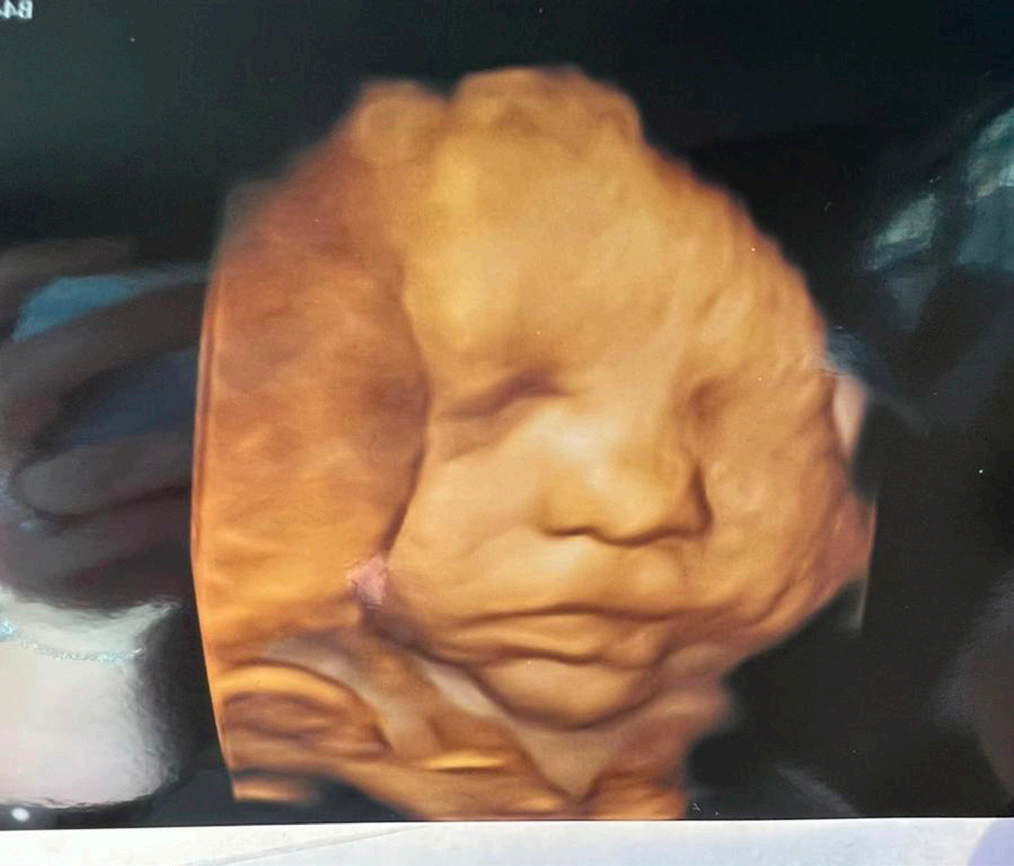
The volume rendered pre-natal ultrasound image performed about 5 weeks before delivery, in the retrospect, depicts facial asymmetry on the right side.

MRI Head, Internal Auditory Meatus and Neck were performed on an Optima MR450w with GEM Suite MR system. The imaging demonstrated congenital absence of the right seventh cranial nerve with normal eighth cranial nerves bilaterally (Figures 1 and 2). The remainder of the cranial nerves, brain parenchyma and parotid glands appeared normal. A 4D ultrasound was performed about 5 weeks prior to delivery, which was privately funded by the parents with no clinical indication. Retrospective review of pre-natal 4D ultrasound images depicts a right downward turn of the mouth, supporting the diagnosis further (Figure 3). This is, to our knowledge, the first published pre-natal ultrasound image of a patient with congenital facial nerve palsy.

## Differential diagnosis

Due to the history of a difficult ventouse delivery, a traumatic cause for the congenital facial palsy was initially considered the primary differential. MR imaging was requested due to the possibility of other causes such as a space occupying lesion or anatomical anomaly. This case highlights the role of MR imaging, as the diagnosis of congenital facial nerve agenesis was confirmed.

## Treatment

Following discussion with parents, it was agreed that the priority for immediate treatment would be an eye lubrication regimen, for protection against exposure keratitis due to difficulty with lid closure on the affected side.

### Outcome and follow up

A diagnosis of isolated congenital facial nerve agenesis was confirmed following MR imaging and multi-disciplinary discussions between ENT and Radiology. Ophthalmology review established that there were no corneal ulcers or visual difficulty, with a plan to discuss lid procedures for eye closure in the near future if needed. A referral for facial physiotherapy was made, and annual follow-up appointments with ENT are planned to monitor progress with growth. The patient is also awaiting an appointment for consideration of nerve reconstruction surgery.

## Discussion

Congenital facial nerve palsy is rare, with an incidence of approximately 0.8–2.3 per 1000 births.^1,2^ It is typically related to injury of the facial nerve following birth trauma, particularly forceps delivery. Traumatic facial nerve palsy as a consequence of forceps delivery has a favourable outcome, demonstrating a recovery rate of up to 89%.^1^ Facial nerve palsy of the newborn from agenesis or hypoplasia is otherwise typically associated with syndromic features. The most well-recognised condition in the literature is Moebius syndrome, which clinically presents with abducens and facial nerve palsies, and has a spectrum of imaging features including the underdevelopment of the facial colliculi.^3^ Further conditions associated with facial nerve palsy include Goldenhaar and CHARGE syndromes, and compressive aetiologies including tumours or vascular malformations. Inflammatory/infective links are also reported, such as Bell’s palsy with human herpes virus.^4,5^

Incidence of isolated congenital nerve agenesis to our knowledge, is limited to very few case reports. The first case was published in 2001, where a 7 year old child with congenital facial palsy underwent parotid surgery for non-tuberculous mycobacterial infection, and an absent ipsilateral facial nerve was discovered incidentally.^6^ The first MR imaging depiction of isolated facial nerve agenesis was published in 2016, presenting two cases of unilateral facial nerve agenesis in 2-month and 6-month-old infants.^7^ Following this, there have been very few case reports of unilateral facial nerve agenesis to date.^8–10^ Additional case reports of unilateral facial nerve agenesis have associations with hearing difficulties and were found to have additional temporal bone or vestibulocochlear nerve pathologies.^11,12^

Confirming the cause for patient’s symptoms has informed further management and prognosis, as facial nerve agenesis carries a less favourable recovery rate than traumatic aetiology. In this case, on examination the patient had no observable facial asymmetry at rest with an intact Bell’s phenomenon, suggesting a degree of aberrant nerve supply to the facial muscles and the possibility of mild clinical improvement, as seen in one previous case study of a child presenting at 6 months old.^7,13^

MR imaging is well-documented and widely recognised as the gold standard diagnostic modality for evaluating the cranial nerves. High-resolution 3D steady-state sequences (*T_2_* weighted) demonstrate detailed images of the cisternal and meatal facial nerve segments, with the low signal nerve contrasted against bright cerebrospinal fluid.^14^ In this case, MR 3D-FIESTA images confirmed congenital absence of the ipsilateral seventh cranial nerve, with similar imaging appearances to previously reported cases also demonstrating unremarkable appearances of the remainder of the brain parenchyma, cranial nerves and parotid glands.^7,8^

Although pre-natal 3D, 4D and 5D ultrasound imaging are not currently routinely available via the National Health Service in the United Kingdom, many patients now choose to self-fund for the increased level of detail it provides. Ultrasound is an imaging modality with increasingly improving resolution with advancements in technology. In this case, the pre-natal ultrasound image further supported the diagnosis of a congenital facial nerve palsy.

## Learning Points

- Although rare, congenital facial nerve agenesis can occur in isolation and can be challenging to diagnose initially, particularly if there is a background of difficult birth history.
- MR imaging remains the gold standard diagnostic modality for distinguishing between traumatic and congenital causes of facial nerve palsy, which has management, prognostic and medio-legal implications.
- Many expectant parents are choosing to self-fund pre-natal 3D, 4D and 5D ultrasound imaging for the increased level of detail it provides. This raises the possibility of identifying congenital facial nerve palsies prenatally in future, or at the very least, strengthen the diagnosis of non-traumatic causes in cases involving birth trauma.
